# Particle Beam Therapy and Surgery as Radical Treatments for Parotid Malignancies—A Single-Center Preliminary Case Study

**DOI:** 10.3390/jcm13113314

**Published:** 2024-06-04

**Authors:** Katsunori Katagiri, Kiyoto Shiga, Daisuke Saito, Shin-Ichi Oikawa, Aya Ikeda, Kodai Tsuchida, Jun Miyaguchi, Takahiro Kusaka, Iori Kusaka, Hisanori Ariga, Ichiro Seto, Tatsuhiko Nakasato, Masashi Koto

**Affiliations:** 1 Department of Head and Neck Surgery, Iwate Medical University School of Medicine, 2-1-1 Idaidori, Yahaba 028-3695, Japan; kats460129@yahoo.co.jp (K.K.); daidaisuke0214@hotmail.com (D.S.); m14_oishin@yahoo.co.jp (S.-I.O.); aikeda@iwate-med.ac.jp (A.I.); k.tsuchida0210@gmail.com (K.T.); miyamj@iwate-med.ac.jp (J.M.); imu.43rd.kusaka@gmail.com (T.K.); strikecosmos@gmail.com (I.K.); 2 Department of Radiology, Iwate Medical University School of Medicine, 2-1-1 Idaidori, Yahaba 028-3695, Japan; hariga2525@gmail.com; 3 Department of Radiation Therapy, Southern TOHOKU Proton Therapy Center, 7-172 Yatsuyamada, Koriyama 963-8052, Japan; setoichiro@gmail.com; 4 Department of Diagnostic Radiology, Southern TOHOKU General Hospital, 7-115 Yatsuyamada, Koriyama 963-8563, Japan; nakasato77@gmail.com; 5 Department of Radiation Therapy, QST Hospital, 4-9-1 Anagawa, Inage-ku, Chiba-shi 263-8555, Japan; koto.masashi@qst.go.jp

**Keywords:** parotid malignancy, particle beam therapy, surgery, treatment outcomes

## Abstract

**Background/Objectives**: Particle beam therapy (PBT) was approved in April 2018 for head and neck malignancies and has since been introduced as a radical therapy for parotid malignancies. However, its prevalence and effectiveness in relation to surgical treatment have not been investigated. **Methods:** In this study, we evaluated 36 patients with parotid malignancy who underwent surgery (n = 26) or PBT (n = 10) and then analyzed the annual changes in the number of patients, survival rates, and clinical factors affecting prognosis. **Results:** Of the ten patients who opted for PBT, two and eight patients underwent PBT before and after 2018, respectively. There was a significant difference between these two groups of patients (*p* = 0.04). Of the ten patients who underwent PBT, five patients were recurrent cases; meanwhile, all twenty-six patients who underwent surgery were receiving initial treatment. Only one patient in each group had local recurrence after the treatment. **Conclusions:** The use of PBT as a radical therapy for parotid malignancies has been increasing since 2018, and patients with recurrent tumors tended to choose PBT. The outcome of the patients who underwent PBT did not seem to be inferior compared with those of the patients who underwent surgery. The histopathological type was a crucial issue in the outcomes of patients who underwent radical therapy for parotid malignancies.

## 1. Introduction

Parotid gland malignancies have various histological variations [1,2], and surgical resection has been considered the first choice of treatment for parotid malignancies [3,4]. However, surgical resection as a radical therapy has some challenges in terms of preservation of facial nerve function, especially in advanced cases and high-grade malignancies, in which cases total parotidectomy is a standard method of surgical resection. Although obtaining negative surgical margins is crucial for preferable patient outcomes [5], those with preoperative facial nerve palsy and/or histological perineural invasion at the time of surgery have been shown to have a worse prognosis [6,7,8]. Particle beam therapy (PBT), which was approved in April 2018 as a treatment for head and neck malignancies, has attracted increasing attention in recent years because of its application in parotid malignancies and its powerful curative effect on tumors [9,10,11,12]. It was reported that both carbon ion beam therapy and proton beam therapy showed good local control rates for patients with various parotid malignancies and were noteworthy for recurrent tumors [9,11]. Patients with parotid malignancies can now select surgery or PBT. Herein, the purpose of this study was to report an analysis of the observable trend of patients choosing parotid malignancy treatment in our hospital and to reveal an increasing application of PBT for patients with parotid malignancies and their outcomes. Although the local control rates of patients who underwent both surgery and PBT as curative treatment were equally good, most of the patients who died of the disease had distant metastases such as lung metastases. Moreover, histopathological type was a crucial issue in determining the outcomes of patients who underwent radical therapy for parotid malignancies.

## 2. Patients and Methods

This retrospective review of patients’ medical records was conducted in accordance with the ethical guidelines of the responsible committee on human experimentation (institutional and national) and adhered to the principles outlined in the Declaration of Helsinki of 1975, as revised in 2008 [13]. Our institutional review board approved this study on March 3, 2021 (MH2020-209). Written informed consent was obtained from all patients.

We enrolled 36 consecutive patients with parotid malignancies who underwent surgery (n = 26) at our hospital or were diagnosed and referred to receive PBT in other hospitals (n = 10) between May 2015 and February 2022. We included the patients who underwent these therapies under radical intent and excluded the patients who underwent palliative therapy. We discussed the eligibility of the patients for these radical therapies at the tumor board including head and neck surgeons and radiologists. The survival rates and clinical factors affecting the prognosis of these patients were examined. The survival rates of the patients were calculated based on the results of the prognostic studies. All patient information was recorded in a digital medical record system and updated whenever a clinical event occurred. The database was regularly updated and preserved.

Our diagnostic procedure before radical therapy consisted of a histopathological study and diagnostic imaging. For patients with parotid tumors, fine-needle aspiration cytology and/or open biopsy under local anesthesia were performed routinely. A histopathological diagnosis was considered for most tumors before radical therapy was initiated. Computed tomography and magnetic resonance imaging were planned regularly for the parotid tumors and neck region. In patients whose tumors were suspected or determined to be malignant, 18-fluoro-deoxy glucose positron emission tomography was performed to determine whether or not the patients had any other malignancies and/or distant metastases.

Our treatment plans for the initial therapy in patients who chose surgical therapy included surgery and postoperative radiotherapy if needed. According to the preoperative diagnosis, for early-stage primary tumors, partial resection of the parotid gland was the preferred and recommended method [4]. For advanced-stage tumors, total or extended total parotidectomy was selected with facial nerve reconstruction. Extended total parotidectomy included mastoidectomy and facial nerve resection at a high (proximal) position and/or partial resection of the mandible and surrounding skin. Neck dissection was added for the preoperative diagnosis of neck lymph node metastases or advanced-stage tumors. Plastic surgeons performed reconstruction surgery, and the most preferred procedure was anterolateral thigh myocutaneous flap reconstruction, followed by rectus abdominis myocutaneous flap reconstruction with facial nerve reconstruction. Postoperative chemoradiotherapy was performed if clinical and pathological assessments indicated a high risk of recurrence, such as a close margin between normal tissues and the tumor or extracapsular invasion of metastatic lymph nodes [14]. The postoperative chemoradiotherapy regimen included administration of docetaxel (50 mg/m^2^) plus CDDP (60 mg/m^2^) every 4 weeks or CDDP (80–100 mg/m^2^) every 3 weeks in combination with radiotherapy (60–70 Gy/30–35 fractions). Intensity-modulated radiotherapy, mainly used as postoperative radiotherapy, was administered daily.

Proton beam therapy was performed at Southern Tohoku Proton Therapy Center, and carbon ion beam therapy was performed at QST Hospital (National Institute of Quantum Science & Technology). The total radiotherapy doses were 67.6–74.8 Gy for the proton beam and 64 Gy for the carbon ion beam. Arterial infusion of CDDP was used as concurrent chemotherapy with proton beam therapy [15]. The total dosage of CDDP ranged from 160 to 480 mg/body. After PBT, the patients were periodically observed at our outpatient clinic, and computed tomography and/or magnetic resonance imaging was regularly conducted.

χ^2^-tests and *t*-tests were used to analyze the annual differences in the cases and clinical factors of the patients. The Kaplan–Meier method was used to evaluate patient survival and local control rates, and a log-rank test was used to examine significant differences between the PBT and surgery cases. Differences were considered statistically significant at *p* < 0.05.

## 3. Results

The histopathology of the parotid tumors is summarized in Table 1. There were ten cases of salivary duct carcinoma, three mucoepidermoid carcinomas (two low-grade and one moderate-grade malignancy), two adenocarcinomas (not otherwise specified [NOS]), two adenoid cystic carcinomas, two epithelial–myoepithelial carcinomas, one squamous cell carcinoma, one myoepithelial carcinoma, one lymphoepithelial carcinoma, one hybrid carcinoma, one secretory carcinoma, one carcinoma ex pleomorphic adenoma, and one angiosarcoma in the surgery group. Among these histopathological types, hybrid carcinoma is a rare type of parotid malignancy. In this case (no. 23), the tumor consisted of salivary duct carcinoma and myoepithelial carcinoma. There were three cases of adenoid cystic carcinoma, three of acinic cell carcinoma, two of salivary duct carcinoma, one of adenocarcinoma NOS, and one of squamous cell carcinoma in the PBT group. Although the differences between the surgery and PBT groups regarding histological type were nonsignificant, there were more salivary duct carcinomas and mucoepidermoid carcinomas in the surgery group and more acinic cell carcinomas in the PBT group. There were eleven female and fifteen male patients in the surgery group and five female and five male patients in the PBT group. The average age of the patients in the surgery and PBT groups was 67.3 and 61.3 years, respectively, and there was a significant difference between these two groups (*p* = 0.02). This means that older patients more frequently underwent surgery. 

**Figure 1:**
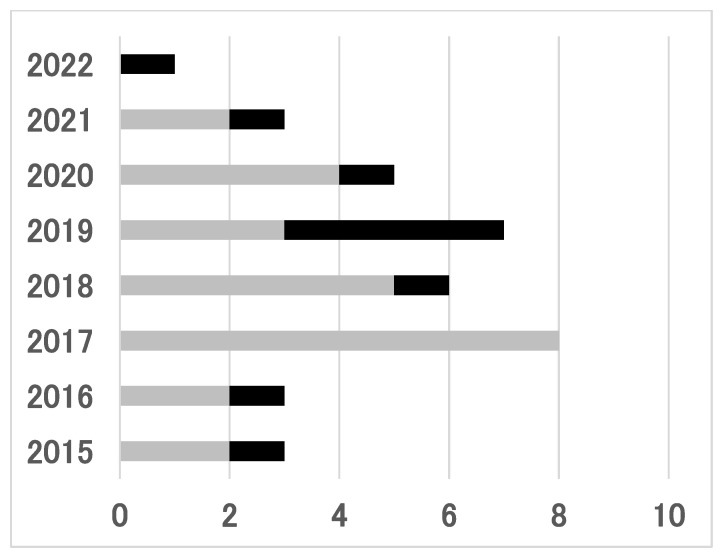
Annual changes in the number of patients with parotid malignancies who underwent surgery and particle beam therapy. Vertical axis, years; horizontal axis, number of patients. Gray, the number of patients who underwent surgery; black, the number of patients who underwent particle beam therapy. Particle beam therapy was approved in April 2018 as a treatment for head and neck malignancies.

Of the ten patients who opted for PBT, two and eight patients underwent PBT before and after 2018, respectively (Figure 1). There was a significant difference between these two groups of patients (*p* = 0.04). After the health insurance system approved PBT for parotid malignancies in 2018, 8 (42%) of 19 patients selected PBT. Notably, 5 of the 10 patients who underwent PBT were recurrent cases (50%). Meanwhile, all of the patients who underwent surgery were receiving initial treatment. There was a significant difference between the surgery and PBT groups (*p* = 0.0001).

Table 2 summarizes the characteristics of patients who underwent PBT as the radical treatment for their tumors. There were one patient with a T1 tumor, two patients with T2 tumors, five patients with T4a tumors, and one patient with a T4b tumor. Lymph node metastases were observed in six patients: one N1, four N2b, and one N3b. Two patients opted for carbon ion beam therapy and eight patients opted for proton beam therapy. Local recurrence was observed in one patient (case no. 3). Three patients died of the disease due to the metastasis, i.e., lung metastasis (case no. 2), multiple metastases (case no. 4), and neck metastases (case no. 6).

Table 3 summarizes the characteristics of patients who underwent surgery as the first-line treatment for their tumors. As local resection procedures, nine patients underwent extended parotidectomy, twelve patients underwent total parotidectomy, four patients underwent superficial parotidectomy, and one patient underwent tumor resection. Eleven patients underwent mastoidectomy to ensure surgical margins in the proximal region of the facial nerve. Five patients underwent skin resection because of skin invasion by the tumor. Neck dissections were performed in 22 patients with lymph node metastases. Sixteen patients underwent facial nerve reconstruction by plastic surgeons. Free flap reconstruction was performed by plastic surgeons in 13 patients, using 11 anterolateral thigh myocutaneous flaps and two rectus abdominis myocutaneous flaps. Postoperative radiotherapy was administered to ten patients: five patients received radiotherapy only, and five patients received radiotherapy with concurrent chemotherapy. The chemotherapy regimens included docetaxel plus CDDP in four patients and CDDP in one patient. Adjuvant chemotherapy was planned for two patients using docetaxel plus CDDP and docetaxel, CDDP, and 5-FU, respectively. Fourteen patients underwent no postoperative therapy, and periodic observation was continued at our outpatient clinic. Six patients died of the disease, all of whom had T4a tumors. Only one patient showed local recurrence, that is, skull base and intracranial tumor invasion. The other four patients showed distant metastases, such as lung, bone, brain, and subcutaneous lesions. One patient (case no. 16) died from multiple neck lymph node metastases. Two patients were too old to visit our outpatient clinic, and their postoperative records ceased. One patient (case no. 6) showed local recurrence and neck metastasis, underwent additional resection surgery and neck dissection 27 months after surgery, and died of another disease 46 months after surgery. In summary, six patients (23% of the patients) died of the disease and the recurrence sites were local recurrence in one, distant metastasis in four, and neck metastasis in one.

**Figure 2:**
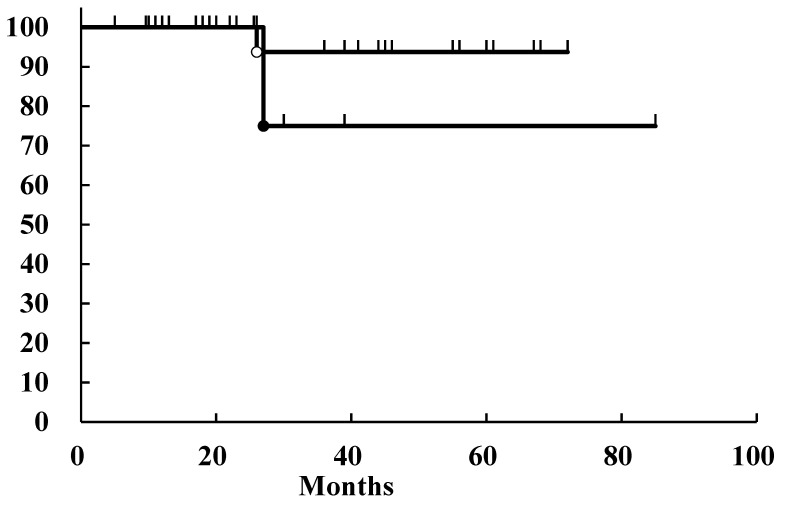
Local control rates of the patients with parotid malignancies. Open circle, patients who underwent surgery; closed circle, patients who underwent particle beam therapy. The 3-year local control rates of the patients in the surgery and PBT groups were 94% and 75%, respectively. There were no significant differences between these two groups according to the log-rank test.

**Figure 3:**
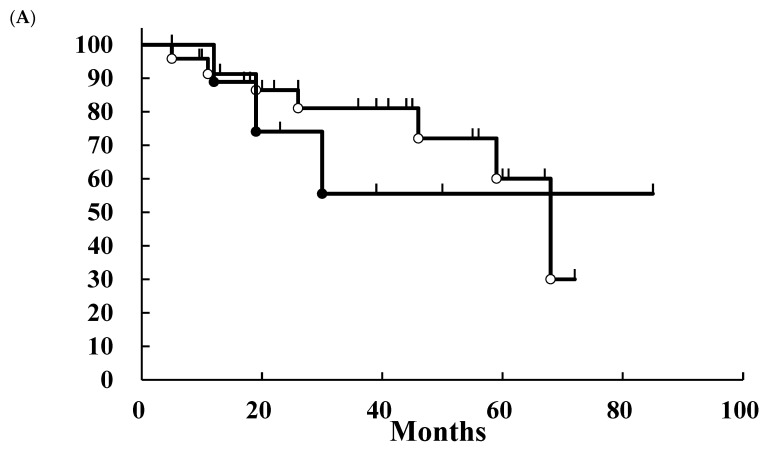

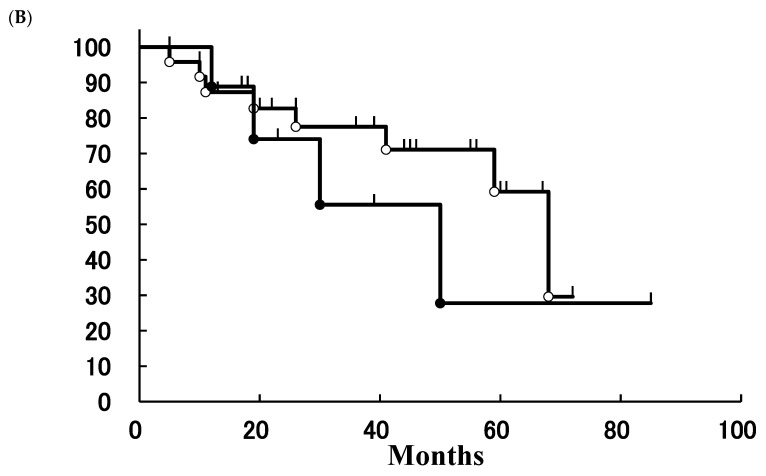
Overall survival (**A**) and disease-free survival (**B**) rates of the patients with parotid malignancies. Open circle, patients who underwent surgery; closed circle, patients who underwent particle beam therapy. The 3-year overall survival rates of the patients in the surgery and particle beam therapy groups were 81% and 56%, respectively. These two groups had no significant differences based on the log-rank test. The 3-year disease-specific survival rates of the patients in the surgery and particle beam therapy groups were 78% and 56%, respectively. These two groups had no significant differences based on the log-rank test.

The outcomes of the patients were analyzed using the Kaplan–Meier method. Only one patient in each group had local recurrence at 26 and 27 months after initial treatment. Therefore, the local control rates (LCRs) of the patients in the surgery and PBT groups were 94% and 75%, respectively (Figure 2). There was no significant difference between these two groups. The overall survival (OS) and disease-free survival (DFS) rates were calculated. The 3-year OS rates of the patients in the surgery and PBT groups were 81% and 56%, respectively (Figure 3A). There was no significant difference between these two groups. The 3-year DFS of the patients in the surgery and PBT groups was 78% and 56%, respectively (Figure 3B). There was no significant difference between these two groups.

## 4. Discussion

Treatment of parotid malignancies has focused on surgical resection because of local control and the shortcomings of other effective treatments. Our study revealed that surgical resection showed a high local control rate (94%) (Figure 2). Only two patients showed local recurrence, and one was salvaged by additional surgery. Distant metastases from parotid malignancies worsen the survival of the treated patients. In our series of patients, four died of metastatic disease. The histopathological types of patients also restricted their survival. In our series of patients, six died of the disease, five of whom had salivary duct carcinomas, and the other patient had an angiosarcoma. Both histological types have extreme malignant potential and poor patient outcomes. These results indicate that our focus must be on treating salivary duct carcinomas to improve the outcomes of patients with parotid malignancies.

Although surgery and postoperative radiotherapy showed favorable PFS and OS [16], the oncologic outcomes of patients worsened when distant metastases appeared. Recently, molecular-targeted therapies such as cetuximab and lenvatinib have been introduced to treat head and neck cancers. The clinicopathological significance of the androgen receptor and HER2 in salicylate duct carcinoma has attracted much attention [17]. Since most salivary duct carcinomas express androgen receptors, and approximately 30% of them also express human epidermal growth factor receptor 2 (Her2) [18], several studies have been carried out to target these molecules [19,20,21,22]. Androgen deprivation therapy (ADT) is the first-line palliative treatment for androgen-positive salivary duct carcinomas, with response rates of 17.6~50% [19,20]. Her2-targeted therapies, such as trastuzumab and docetaxel, have been shown to have remarkable effects in patients with Her2-positive salivary duct carcinoma [21,22]. These therapies are recommended by the latest National Comprehensive Cancer Network (NCCN) [23] and American Society for Clinical Oncology (ASCO) [4] guidelines. However, these guidelines have demonstrated no preferred regimens, and no single agent or combination therapy has been shown to have a survival advantage. Therefore, further studies are needed to elucidate the recommended therapy for ADT and Her2-targeted therapy.

Why do most patients with salivary duct carcinoma undergo surgery, and fewer patients undergo PBT? In our series of patients who underwent surgery, 10 had salivary duct carcinoma, and 7 of them had multiple neck lymph node metastases at diagnosis. Because of the low effectiveness of radiation therapy and chemotherapy, the head and neck surgeons first selected surgical treatment, including local resection of the primary tumor and neck dissection, for patients with parotid malignancy and neck metastases as an initial treatment. This tendency could affect the proportion of patients with salivary duct carcinoma in the surgery and PBT groups, that is, only two patients with salivary duct carcinoma in the PBT group.

The selection of PBT as a curative treatment for parotid malignancies has increased in recent years. It is assumed that this tendency reflected the timeframe of approval of PBT for parotid malignancies from the health insurance system of Japan in 2018. Patients who had parotid malignancies and were indicated for parotidectomy (partial, total, and extended total) were dissatisfied because of loss of facial nerve function after surgery. If patients showed complete response after PBT, their facial nerve function would be preserved whether they had total or partial facial nerve function during pretreatment. Considering the tendencies for PBT preference, the number of patients with parotid malignancies who undergo PBT will increase in the future.

Our study revealed that patients with recurrent tumors were inclined to choose PBT. In fact, previous reports on PBT included recurrent cases of parotid malignancies, and they had fair results [10,11,12]. In our series of patients, five with recurrent tumors underwent PBT, and three were alive without disease. These results were compatible with previous reports [10,11,12], suggesting the usefulness of PBT for recurrent tumors of parotid malignancies.

As for the outcomes of the patients, our study was preliminary in a single institution, and surgery and PBT could not be compared directly because of the distribution of the patients in both groups, such as recurrent cases. However, our study also revealed that the outcome of the patients who underwent PBT did not seem to be inferior to those of patients who underwent surgery. Although the number of patients who underwent PBT was much smaller than those who underwent surgery, making the calculated rate of patients appear worse, the LCRs of PBT were almost equal to that of surgery. Furthermore, the OS and DFS of the patients who underwent PBT were not much worse than those who underwent surgery, but half of the patients who underwent PBT had recurrence.

Koto et al. reported that the 5-year LCR and OS of the 46 patients with locally advanced parotid cancer, including 21 recurrent cases who underwent carbon ion beam therapy, were 74.5% and 70.1%, respectively [9]. Their patients included sixteen cases of adenoid cystic carcinoma, eight of mucoepidermoid carcinoma, and eight of adenocarcinoma NOS, eight T4a and sixteen T4b tumors. Hayashi et al. reported the results of the J-CROS 1402 HN multi-institutional clinical research using carbon ion beam therapy [11]. The 3-year LCR and OS of 58 patients with parotid cancers, including ACC (48%), mucoepidermoid carcinoma (14%), T4 (48%), and unresectable carcinoma (43%), were 81% and 91%, respectively. Meanwhile, Azami et al. reported the results of proton beam therapy for patients with recurrent parotid malignancies [10]. Half of the patients were treated with the concurrent use of intra-arterial infusion of CDDP (250~500 mg/body). The 3-year LCR and OS of their patients, including six cases of T4a tumor and one case of T4b tumor, were 60% and 60%, respectively. Zakeri et al. reported the results of 68 patients with major salivary duct malignancies, including 51 patients with parotid malignancies who underwent proton beam therapy at Memorial Sloan Kettering Cancer Center [12]. In total, 19.1% of the patients were treated with concurrent chemotherapy, mainly CDDP. The 3-year LCR, DFS, and OS of their patients were 95.1%, 80.7%, and 96.1%, respectively. The LCR (75%), DFS (56%), and OS (56%) of the patients in our PBT group were compatible with these previous studies.

Despite the histopathological variety of the parotid malignancies, previous reports using PBT showed good LCR and OS in the treatment of parotid malignancies, especially for advanced and/or recurrent cases. In this study, the 3-year LCR, DFS, and OS of the patients who underwent PBT were 75%, 56%, and 56%, respectively. Because half of our patients were recurrent cases, the DFS and OS were not very good. However, their LCR was compatible with those of previous reports. Our results indicated that PBT could be useful for patients with advanced and/or recurrent parotid malignancies. In addition, our results indicated that surgical resection showed substantially reasonable local control in our series of patients with parotid malignancies, perhaps through effective resection procedures, including extended total parotidectomy and mastoidectomy, to ensure sufficient surgical margins. Radical or prophylactic therapy for metastatic disease is needed in patients with parotid malignancies expected to have a better prognosis. As salivary duct carcinoma is frequently the worst histopathological type of parotid malignancy, additional therapies are needed or developed for this type of parotid malignancy, such as ADT, Her2-targeted therapy, and so on. 

This study has some limitations. This retrospective study included a relatively small number of patients, especially patients who underwent PBT. In addition, it was performed in a single center, which can limit the generalizability of the findings. However, despite these limitations, considerable results were obtained regarding the good LCR of the patients who underwent PBT, despite the fact that the PBT group included many recurrent cases. This report also showed good LCR of the patients who underwent surgery and the limitation of the treatment because most of the patients died of disease with distant metastases. Future studies encompassing large patient cohorts and/or prospective approaches may reveal more specific results for PBT.

## 5. Conclusions

Our study suggested that the use of PBT as a radical therapy for parotid malignancies has been increasing since 2018, and patients with recurrent tumors tended to choose PBT. The outcome of the patients who underwent PBT did not seem to be inferior compared with those of the patients who underwent surgery. The histopathological type was a crucial issue in the outcomes of patients who underwent radical therapy for parotid malignancies.

## Figures and Tables

**Table 1:** Characteristics of the patients.

	Surgery (n = 26)	Particle Beam Therapy (n = 10)	*p*-Value
Average age (years)	67.3	61.3	0.02
Sex (female/male)	11/15	5/5	0.67
Histopathology			
Salivary duct carcinoma	10	2	
Adenoid cystic carcinoma	2	3	
Adenocarcinoma, not otherwise specified	2	1	
Mucoepidermoid carcinoma	3	0	
Acinic cell carcinoma	0	3	
Squamous cell carcinoma	1	1	
Epithelial–myoepithelial carcinoma	2	0	
Myoepithelial carcinoma	1	0	
Lymphoepithelial carcinoma	1	0	
Hybrid carcinoma	1	0	
Secretory carcinoma	1	0	
Carcinoma ex pleomorphic adenoma	1	0	
Angiosarcoma	1	0	
T classification			
T1	0	1	T1, T2 vs. T3, T4
T2	7	2	0.85
T3	7	1	
T4a	12	4	
T4b	0	2	
N classification			
N0	13	4	N0 vs. N+
N1	2	1	0.59
N2b	8	4	
N3b	3	1	
Initial treatment	26	5	0.0001
Recurrent tumor	0	5	

hybrid ca., salivary duct carcinoma + myoepithelial carcinoma.

**Table 2:** Characteristics of the patients who underwent particle beam therapy.

Patient No.	Age (Years)	Sex	Histopathology	TNM	Treatment (Type and Dose)	Outcome
1	67	Male	Acinic cell carcinoma	rT4bN1M0	CIB, 64 Gy	AwoD, 15M
2	56	Female	Adenocarcinoma	rT2N2bM0	PB, NS	DOD, 15M
3	70	Female	Squamous cell carcinoma	rT4aN2bM0	PB, 70 Gy	AWD, 18M
4	45	Male	Adenoid cystic carcinoma	T4bN3bM0	PB, 67.6 Gy	DOD, 18M
5	49	Female	Acinic cell carcinoma	rT2N0M0	PB, 70.4 Gy	AwoD, 23M
6	63	Male	Salivary duct carcinoma	T4aN2bM0	CIB, 64 Gy	DOD, 30M
7	67	Female	Acinic cell carcinoma	rT1N0M0	PB, 70.4 Gy	AwoD, 39M
8	81	Male	Salivary duct carcinoma	T4aN0M0	PB, 70.2 Gy	AwoD, 18M
9	67	Female	Adenoid cystic carcinoma	T4aN2bM0	PB, 74.8 Gy	AwoD, 17M
10	54	Male	Adenoid cystic carcinoma	T3N0M0	PB, 74.8 Gy	AwoD, 5M

CIB, carbon ion beam; PB, proton beam; NS, not specified; AwoD, alive without disease; DOD, died of disease; AWD, alive with disease; M, months.

**Table 3:** Characteristics of the patients who underwent surgery.

No	Age	Sex	Histopathology	cTNM	Surgery	POCRT	Surgical Margin	Lymph Node Metastasis	Outcome
1	77	M	SDC	T4aN2b	TP + M + ND + ALT + NR	D/C + 60Gy	−	18/33 II, III, IV, V	AwoD, 72M
2	81	M	angiosarcoma	T4aN2b	ETP + M + ND + ALT + NR	D/C/F	+	4/12 II, III	DOD, 5M
3	64	M	SDC	T2N2b	SP + ND	D/C + 60Gy	-	5/46 I, II, IV pN3b	AwoD, 45M
4	78	F	adenocarcinoma	T2N0	ETP + M + ND + ALT + NR	-	-	-	AwoD, 67M
5	65	M	SDC	T4aN2b	ETP + M + ND + ALT + NR	D/C + 66Gy	-	4/15 II pN3b	DOD, 68M
6	72	M	adenocarcinoma	T3N0	SP + ND	-	+	1/5 paraparotid pN1	DOOD, 46M
7	75	M	SDC	T4aN2bM1	TP + M + ND + NR	D/C	+	11/23 II, III, IV pN3b	DOD, 9M
8	64	M	SDC	T2N0	TR	60Gy	-	-	AWD, 41M
9	78	M	SCC	T3N2b	ETP + M + ND + ALT + NR	-	-	7/25 II, IV	AwoD, 55M
10	73	F	myoepithelial ca.	T4aN0	TP	66Gy	+	-	AwoD, 61M
11	51	F	MEC intermediate	T2N0	TP + ND + NR	60Gy	-	-	AwoD, 60M
12	64	M	SDC	T4aN0	TP + ND + NR	-	+	-	DOD, 59M
13	57	F	MEC low	T4aN1	ETP + M + ND + ALT + NR	-	-	-	AwoD, 56M
14	86	F	SDC	T4aN0	TP + M + ND	-	-	-	N.D.
15	69	M	SDC	T4aN0	ETP + SR + ND + ALT + NR	-	-	-	DOD, 26M
16	79	F	SDC	T4aN3b	TP + SR + ND + ALT + NR	-	-	58/89 I, II, III, IV contra II pN3b	DOD, 11M
17	39	M	secretary ca.	T2N0	TP + SR + ND + ALT + NR	-	-	2/24 II, III pN2b	AwoD, 36M
18	48	F	ACC	T3N0	ETP + M + ND + ALT + NR	66Gy	-	-	AwoD, 44M
19	65	F	ACC	T2N0	SP	-	-	-	AwoD, 13M
20	60	F	MEC low	T3N0	ETP + M + ND + ALT + NR	-	+	-	AwoD, 39M
21	60	M	SDC	T3N3b	ETP + SR + M + ND + RAMC	C + 60Gy	+	37/42 I, II, III, IV pN3b	AwoD, 26M
22	82	M	epith-myoepi ca.	T2N1	TP + M + ND + NR	-	+	1/48 paraparotid	AwoD, 22M
23	62	M	hybrid ca.	T4aN3b	SP + SR + RAMC	D/C + 66Gy	+	8/47 I, II, IV pN3b	AWD, 10M
24	82	F	epith-myoepi ca	T4aN2b	TP + MR + ND	-	-	3/33 I	N.D.
25	69	M	Ca. ex PMA	T3N0	TP + ND	-	-	-	AwoD, 20M
26	60	F	lymphoepithelial ca.	T3N2b	TP + ND + NR	66Gy	+	26/67 I, II, III, V pN3b	AwoD, 10M

POCRT, postoperative chemoradiotherapy; SDC, salivary duct carcinoma; SCC, squamous cell carcinoma; ca., carcinoma; MEC intermediate, mucoepidermoid carcinoma, intermediate-grade malignancy; MEC low, mucoepidermoid carcinoma, low-grade malignancy; ACC, adenoid cystic carcinoma; epith-myoepi ca., epithelial–myoepithelial carcinoma; hybrid ca., salivary duct carcinoma + myoepithelial carcinoma; Ca. ex PMA, carcinoma ex pleomorphic carcinoma; TP, total parotidectomy; M, mastoidectomy; ND, neck dissection; ALT, anterolateral thigh myocutaneous flap reconstruction; NR, nerve reconstruction; ETP, extended total parotidectomy; SP, superficial parotidectomy; TR, tumor resection; SR, skin resection; RAMC, rectus abdominis myocutaneous flap reconstruction; MR, mandibular bone resection; D, docetaxel; C, CDDP; F, 5-FU; AwoD, alive without disease; DOD, died of disease; DOOD, died of other diseases; AWD, alive with disease; M, months; N.D., not determined.

## Data Availability

The data presented in this study are available on request from the corresponding author. The data are not publicly available due to privacy or ethical restrictions.
